# Purification and Characterization of Biofilm-Associated EPS Exopolysaccharides from ESKAPE Organisms and Other Pathogens

**DOI:** 10.1371/journal.pone.0067950

**Published:** 2013-06-21

**Authors:** Patrick M. Bales, Emilija Miljkovic Renke, Sarah L. May, Yang Shen, Daniel C. Nelson

**Affiliations:** 1 Institute for Bioscience and Biotechnology Research, University of Maryland, Rockville, Maryland, United States of America; 2 Department of Veterinary Medicine, University of Maryland, College Park, Maryland, United States of America; Loyola University Medical Center, United States of America

## Abstract

In bacterial biofilms, high molecular weight, secreted exopolysaccharides can serve as a scaffold to which additional carbohydrates, proteins, lipids, and nucleic acids adhere, forming the matrix of the developing biofilm. Here we report methods to extract and purify high molecular weight (>15 kDa) exopolysaccharides from biofilms of eight human pathogens, including species of *Staphylcococcus*, *Klebsiella*, *Acinetobacter*, *Pseudomonas*, and a toxigenic strain of *Escherichia coli* O157:H7. Glycosyl composition analysis indicated a high total mannose content across all strains with *P. aeruginosa* and *A. baumannii* exopolysaccharides comprised of 80–90% mannose, *K. pneumoniae* and *S. epidermidis* strains containing 40–50% mannose, and *E. coli* with ∼10% mannose. Galactose and glucose were also present in all eight strains, usually as the second and third most abundant carbohydrates. N-acetyl-glucosamine and galacturonic acid were found in 6 of 8 strains, while arabinose, fucose, rhamnose, and xylose were found in 5 of 8 strains. For linkage analysis, 33 distinct residue-linkage combinations were detected with the most abundant being mannose-linked moieties, in line with the composition analysis. The exopolysaccharides of two *P. aeruginosa* strains analyzed were consistent with the Psl carbohydrate, but not Pel or alginate. The *S. epidermidis* strain had a composition rich in mannose and glucose, which is consistent with the previously described slime associated antigen (SAA) and the extracellular slime substance (ESS), respectively, but no polysaccharide intracellular adhesion (PIA) was detected. The high molecular weight exopolysaccharides from *E. coli*, *K. pneumoniae*, and A. *baumannii* appear to be novel, based on composition and/or ratio analysis of carbohydrates.

## Introduction

Microorganisms that infect humans differ in mechanisms of pathogenesis, virulence factors, and antimicrobial resistance profiles. However, one common trait shared by most is the ability and propensity to form biofilms [1]. Along with upregulation of adhesins, phenotypic changes cause bacteria to secrete high molecular weight exopolysaccharides during conversion from planktonic to biofilm modes of growth [2]–[5]. These exopolysaccharides can make up a crucial part of the extracellular polymeric substance (EPS) associated with biofilm development that serves to cement whole bacterial populations to a surface rather than enclosing individual cells [6]. Also included in the EPS are proteins, secreted nucleic acids, humic substances, and metal ions. Together, the EPS protects biofilm bacteria from environmental stress [7], [8].

The role of the EPS in pathogenesis has been studied in many organisms where the biofilm mode of growth has been shown to allow for increased resistance to antibiotic treatment, the immune response, and nutrient-limiting conditions within the host, promoting long-term persistence [9]. Biofilm formation on medical implant devices such as catheters and mechanical heart valves is also a major problem that is closely tied to the adhesion- and resistance-related abilities granted them by the ability to synthesize and secrete exopolysaccharides [10]–[12]. Human pathogens associated with biofilm development include species of *Enterococcus faecalis, Staphylococcus aureus*, *Klebsiella pneumoniae*, *Acinetobacter baumannii*, *Pseudomonas aeruginosa*, and *Enterobacter spp*. These “ESKAPE” pathogens are the leading causes of nosocomial infections [13], [14] and are so-named to emphasize their ability to “escape” the effects of antimicrobial treatment due to acquisition of resistance genes as well as formation of biofilms.

While surface-associated exopolysaccharides and capsules play a role in both extracellular and intracellular adherence during the conversion from planktonic to biofilm growth, our interests focus on the secreted exopolysaccharides, particularly the high molecular weight exopolysaccharides that are believed for form the “backbone” of the EPS to which proteins, nucleic acids, and capsular polysaccharides adhere [6], [15]. While there are many protocols in the literature for isolation of capsular polysaccharides or for the extraction of total bacterial EPS, only a few have attempted to fractionate exopolysaccharides by size [16] and none have been specifically tailored for the isolation of high molecular weight backbone exopolysaccharides from biofilms. Bulk EPS extraction requires methods that physically break up the biofilm matrix such as ultrasonication or EDTA, which promotes EPS separation by chelating cations that are thought to crosslink polysaccharide chains within the EPS. The use of glutaraldehyde or formaldehyde is also common to fix bacterial cells to prevent contamination via cell lysis during the extraction steps [17], [18]. Once the bulk EPS is extracted, the polysaccharide fraction must be separated from DNA, proteins, and lipids. Oliveira and colleagues successfully utilized 20% trichloroacetic acid (TCA) for the precipitation of proteins from the EPS [19], whereas Sutherland used ethanol to precipitate the polysaccharide fraction [20]. Similar protocols have also included the use of NaOH to promote dissociation of acid groups within the EPS for increased solubility, cation exchange chromatography, differential centrifugation, and selective dialysis [9], [21]–[23]. Our laboratory has combined portions of the above protocols and added a size exclusion chromatography step to produce an effective method of purifying high molecular weight EPS exopolysaccharides. We then apply this methodology to biofilms of eight medically important pathogens, including several representative ESKAPE organisms, as well as a methicillin-resistant *Staphylococcus epidermidis* strain and a toxigenic strain of *Escherichia coli* O157:H7, both of which are known biofilm producers. Finally, we characterized the resulting EPS exopolysaccharides by composition and linkage analysis.

## Materials and Methods

### Bacterial strains and growth conditions

Unless otherwise indicated, all reagents were purchased from Thermo-Fisher Scientific and were of the highest purity available. *S. epidermidis* strain NRS-101 was obtained from the Network on Antimicrobial Resistance in *S. aureus* (NARSA). All other strains were purchased from the American Type Culture Collection (ATCC). These include two *P. aeruginosa* strains, 700829 and 700888, both known biofilm production strains; two *K. pneumoniae* strains, 700603, a multi-drug resistant strain and 700831, a biofilm strain; two *A. baumannii* strains, BAA-1878 and BAA-1605, a multi-drug resistant strain; and one *E. coli* strain, 43894, a toxigenic O157:H7 serotype. *S. epidermidis* strain NRS101 was grown in brain-heart infusion media, *K. pneumoniae* strain 700603 was grown in Luria broth, and all other strains were grown in tryptic soy broth. Glycerol stocks of all strains were stored at −80°C. Biofilms grown for EPS purification were prepared by inoculating 20 ml of overnight culture into 400 ml of fresh media in a 1.5 L Fernbach flask to provide a large surface area for biofilm adherence. Biofilms were grown at 37°C without shaking for 4–5 days until a thick biofilm “sludge” was observed.

### EPS extraction

After development of a mature biofilm, 60 µl of formaldehyde (36.5% solution) was added to each 10 ml of sludge to fix the cells and prevent cell lysis during subsequent steps. The formaldehyde-sludge mixture was incubated at room temperature in a chemical hood with gentle shaking (100 rpm) for 1 hour. Four ml of 1 M NaOH was added for each 10 ml of sludge and incubated at room temperature, with shaking, for 3 hours to extract EPS. Cell suspensions were then centrifuged (16,800×g) for 1 hour at 4°C. The supernatant containing soluble EPS was filtered through a 0.2 µm filter (Corning) and dialyzed against distilled water using a 12–14 kDa molecular weight cut-off (MWCO) membrane for 24 hours at 25°C.

### Purification of exopolysaccharides

TCA was added (20% w/v) to extracted EPS solutions on ice to precipitate proteins and nucleic acids. After 30 minutes, the solution was centrifuged (16,800×g) for 1 hour at 4°C, the supernatant was collected, and 1.5 volumes of 95% ethanol was added and the mixture was placed at −20°C for 24 hours to precipitate exopolysaccharides away from lipids. The solution was then centrifuged (16,800×g) for 1 hour at 4°C and the exopolysaccharide pellet was resuspended in Milli-Q water and dialyzed against the same for 24 hours at 4°C using a 12–14 kDa MWCO membrane to remove low molecular weight impurities and the remaining retentate was lyophilized overnight. The lyophilized powder was then resuspended in 5–10 ml of phosphate buffered-saline (pH 7.4) and purified on a 26/60 S-200 gel filtration column (GE Healthcare) using an AKTA FPLC system (GE Healthcare) that had been calibrated with gel filtration standards (Bio-Rad) to generate a standard curve of apparent molecular mass vs. retention volume. Fractions were tested for the presence of carbohydrates by the phenol-sulfuric acid method of DuBois as previously described [24] and only high molecular weight fractions, defined as >15 kDa, containing carbohydrates were pooled, dialyzed against Milli-Q water to remove PBS, and lyophilized a final time for subsequent composition and linkage analysis.

### Composition and linkage analysis

Carbohydrate composition and linkage analysis was performed at the Complex Carbohydrate Research Center (Athens, GA) as previously described [25], [26]. Briefly, for glycosyl composition analysis, an aliquot (∼500 µg) was taken from the purified EPS exopolysaccharide sample and added to a separate tube with 20 µg of inositol as an internal standard. Methyl glycosides were then prepared from the dry sample by methanolysis in 1 M HCl in methanol, followed by re-*N*-acetylation with pyridine and acetic anhydride in methanol for detection of amino sugars. The sample was then per-*O*-trimethylsilylated (TMS) by treatment with Tri-Sil (Pierce). Combined gas chromatography/mass spectrometry (GC/MS) analysis of the TMS methyl glycosides was performed on an Agilent 6890N GC interfaced to a 5975B MSD (mass selective detector), using a Supelco EC-1 fused silica capillary column (30 m×0.25 mm ID). For glycosyl linkage analysis, an aliquot (∼500 µg) was taken from the purified EPS exopolysaccharide sample and suspended in ∼300 µl of dimethyl sulfoxide and placed on a magnetic stirrer for 2 days. The sample was then permethylated by the method of Ciukanu and Kerek [27], hydrolyzed for 2 hours with 2 M trifluoroacetic acid in a sealed tube at 121°C, reduced with NaBD_4_, and acetylated using acetic anhydride/pyridine. The resulting partially methylated alditol acetates were analyzed by GC/MS as described above.

### Biofilm microscopy

Biofilms were grown in two-well chamber slides (Lab-Tek) with 1 ml of tryptic soy broth for 1 or 3 days. Biofilm wells were washed 2X with PBS and then stained with 5 µg of the FITC-labeled Hippeastrum hybrid lectin (HHA) from Amaryllis (EY Labs) and Hoechst 33342, a nucleic acid stain (Invitrogen), in PBS for 1 hour at room temperature. After incubation, the wells were again washed 2X with PBS, chambers were removed from the glass slide, and biofilms attached to the slides were imaged by an Eclipse 80i fluorescent microscope workstation (Nikon) or an LSM710 laser scanning confocal microscope workstation (Zeiss) as previously described [28]. NIS-Elements (Nikon) or ZEN (Zeiss) software packages were used for image analysis.

### Results and Discussion

### EPS purification

**Figure 1:**
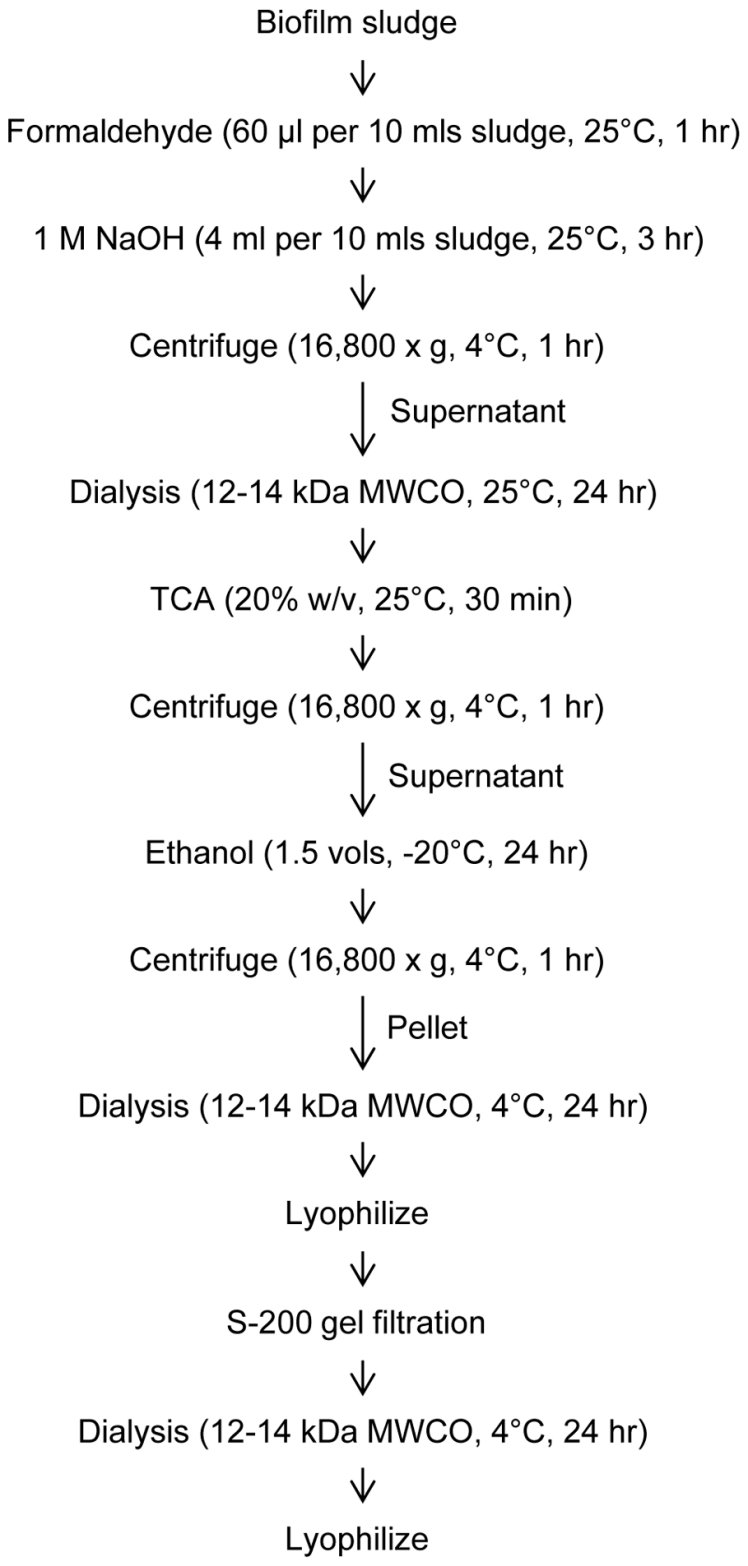
Schematic for extraction and purification of EPS exopolysaccharides. Depending on strain, yields range from 2–15 mg of purified polysaccharide per starting 1.2 L of sludge.

A schematic of the protocol we developed to purify EPS exopolysaccharides from biofilms is shown in **Figure 1**. This methodology incorporates into one protocol many extraction and purification steps successfully demonstrated by others [17]–[23], along with selection steps for high molecular weight for exopolysaccharides (i.e.>15 kDa) through use of large pore dialysis and gel filtration. As detailed by specific examples in the sections below, our protocol did not result in isolation of surface-associated or capsular polysaccharides, indicating that the methods were specific for secreted exopolysaccharides. Additionally, the protocol was robust, allowing us to successfully extract EPS exopolysaccharide from all eight bacterial strains representing five species. Our yields ranged from 2–15 mg of purified EPS exopolysaccharide for each strain from 1.2 L of biofilm culture (three Fernbach flasks, each containing 400 ml of bacteria). This is lower than yields reported by other methods, but is most likely due to our selection of only the high molecular weight exopolysaccharide that forms the EPS backbone, which excludes lower molecular weight exopolysaccharides and oligosaccharides that would co-purify with more crude purification methods.

### Composition and linkage analysis

The glycosyl composition results are summarized in **Table 1**. Across all samples, a total of 11 sugars and aminosugars were detected. Notably, mannose was found in exopolysaccharides of all strains and was the predominant carbohydrate in every strain except *E. coli*. Indeed, mannose accounted for ∼80–90% of the total carbohydrate content in *P. aeruginosa* and *A. baumannii* species and ∼40–50% of the total carbohydrate in *K. pneumoniae* and *S. epidermidis* species. Galactose and glucose were also found in all eight strains tested and were often ranked as the second or third most abundant carbohydrate. Other carbohydrates were also found to be well represented in biofilm EPS exopolysaccharides. *N*-acetyl-glucosamine (GlcNAc) and galacturonic acid (GalA) were found in 6 of 8 strains while arabinose, fucose, rhamnose, and xylose were found in 5 of 8 strains. In contrast, *N*-acetyl-galactosamine (GalNAc) was only present in the *E. coli* and *S. epidermidis* strains and glucuronic acid (GlcA) was only found in a single *K. pneumoniae* strain.

**Table 1:** Glycosyl Composition Analysis.

Glycosyl Residue	Abbreviation	*P. aeruginosa*		*K. pneumoniae*		*A. baumannii*		*S. epidermidis*	*E. coli*	Frequency
		700829	700888	700603	700831	BAA-1605	BAA-1878	NRS 101	43894	
Arabinose	Ara	1.6	2.4	–	1.5	1.1	0.5	–	–	5
Fucose	Fuc	0.2	0.2	–	0.3	0.1	–	–	22.6	5
Galactose	Gal	3.0	3.7	14.0	7.8	4.0	13.2	1.7	2.1	8
Galacturonic Acid	GalA	0.7	0.8	2.5	9.8	2.0	1.0	–	–	6
Glucose	Glc	3.6	6.8	1.3	31.1	7.9	4.5	35.7	36.8	8
Glucuronic Acid	GlcA	–	–	5.0	–	–	–	–	–	1
Mannose	Man	89.5	84.3	49.4	38.3	84.0	79.3	52.8	9.8	8
*N*-Acetyl-Galactosamine	GalNAc	–	–	–	–	–	–	6.3	26.8	2
*N*-Acetyl-Glucosamine	GlcNAc	–	–	0.8	0.7	0.4	1.3	3.5	1.9	6
Rhamnose	Rha	1.0	1.3	27.0	10.0	0.1	–	–	–	5
Xylose	Xyl	0.4	0.5	–	0.5	0.4	0.2	–	–	5
Total:		100.0	100.0	100.0	100.0	100.0	100.0	100.0	100.0	

Values expressed as mole percent of total carbohydrate.

–, not detected.

The glycosyl linkage analysis for EPS exopolysaccharide of all strains is summarized in **Table 2**. Across all samples, 33 distinct residue-linkage combinations were detected. Consistent with the glycosyl composition results, a substantial proportion of the total linkages involved mannose. 2-linked mannose (2-Man) and terminally-linked mannose (t-Man) residues were detected in all samples, whereas 3-linked mannose (3-Man), 6-linked mannose (6-Man), and 4-linked glucose (4-Glc) were present in the EPS exopolysaccharide of 7 of the 8 strains tested. Mannose is also a common branch point for these complex structures as 2,6-linked mannose (2,6-Man) was present in 6 strains, 2,3-linked mannose (2,3-Man) was present in five strains, 3,6-linked mannose (3,6-Man) was present in three strains, and 2,3,4-linked mannose (2,3,4-Man) was present in one strain.

**Table 2:** Glycosyl Linkage Analysis.

Glycosyl Linkage	Abbreviation	*P. aeruginosa*		*K. pneumoniae*		*A. baumannii*		*S. epidermidis*	*E. coli*	Frequency
		700829	700888	700603	700831	BAA-1605	BAA-1878	NRS 101	43894	
4-linked arabinopyranosyl residue	4-Ara	0.2	0.2	–	0.2	–	0.2	–	–	4
3-linked fucopyranosyl residue	3-Fuc	–	–	–	–	–	–	–	3.6	1
terminally-linked fucopyranosyl residue	t-Fuc	–	–	–	–	–	–	–	0.9	1
3-linked galactopyranosyl residue	3-Gal	–	0.3	18.2	–	–	0.3	–	–	3
3,6-linked galacopyranosyl residue	3,6 Gal	0.3	0.2	–	–	0.8	0.2	–	–	4
4-linked galactopyranosyl residue	4-Gal	1.2	1.6	–	0.2	1.1	1.6	–	1.1	6
terminally-linked galactopyranosyl residue	t-Gal	0.1	0.2	0.3	–	–	0.2	1.5	0.2	6
4-linked *N*-acetyl-galactosamine	4-GalNAc	–	–	–	–	–	–	–	0.1	1
6-linked *N*-acetyl-galactosamine	6-GalNAc	–	–	–	–	–	–	–	2.8	1
terminally-linked *N*-acetyl-galactosamine	t-GalNAc	–	–	–	–	–	–	–	0.4	1
2-linked glucopyranosyl residue	2-Glc	–	–	–	–	–	–	6.5	–	1
2-linked 6-deoxy-4 glucosamine	2-(6-deoxy)-4-GlcN	–	–	–	–	–	–	–	13.0	1
3-linked glucopyranosyl residue	3-Glc	1.2	1.3	–	14.4	–	1.3	4.0	–	5
3,6-linked glucopyranosyl residue	3,6-Glc	–	–	–	–	–	0.7	1.3	–	2
4-linked glucopyranosyl residue	4-Glc	0.2	0.4	0.4	0.4	–	0.4	1.2	39.2	7
4,6-linked glucopyranosyl residue	4,6-Glc	–	–	–	11.4	–	–	–	–	1
6-linked glucopyranosyl residue	6-Glc	0.6	0.7	–	0.4	1.3	0.8	1.3	–	6
terminally-linked glucopyranosyl residue	t-Glc	–	–	0.0	1.2	–	–	3.7	6.3	3
4-linked *N*-acetyl-glucosamine	4-GlcNAc	–	–	–	–	–	–	–	0.2	1
6-linked *N*-acetyl-glucosamine	6-GlcNAc	–	–	–	–	–	–	–	3.3	1
terminally-linked *N*-acetyl-glucosamine	t-GlcNAc	–	–	–	–	–	–	–	0.9	1
2-linked hexafuranosyl residue	2-HexF	–	–	–	12.2	–	–	–	–	1
2-linked mannopyranosyl	2-Man	20.6	19.3	40.1	13.8	20.2	19.2	6.0	2.0	8
2,3-linked mannopyranosyl residue	2,3-Man	1.3	1.2	15.4	0.5	–	1.2	–	–	5
2,3,4-linked mannopyranosyl residue	2,3,4-Man	–	–	–	11.6	–	–	–	–	1
2,6-linked mannopyranosyl residue	2,6-Man	32.3	28.5	–	16.1	32.5	28.5	32.3	–	6
3-linked mannopyranosy lresidue	3-Man	16.7	16.0	15.4	5.2	16.5	15.9	7.5	–	7
3,6-linked mannopyranosyl residue	3,6-Man	0.6	0.7	–	0.4	–	–	–	–	3
4-linked mannopyranosyl residue	4-Man	–	0.4	–	–	–	0.4	1.2	–	3
6-linked mannopyranosyl residue	6-Man	1.4	1.5	–	1.2	2.2	1.6	5.7	0.7	7
terminally-linked mannopyranosyl residue	t-Man	23.3	27.4	1.3	10.5	25.4	27.4	27.8	25.3	8
2-linked rhamnopyranosyl residue	2-Rha	–	–	7.3	–	–	–	–	–	1
terminally-linked rhamnopyranosyl residues	t-Rha	–	–	1.6	0.3	–	–	–	–	2
2-linked xylopyranosyl residue	2-Xyl	–	0.1	–	–	–	0.1	–	–	2
Total:		100.0	100.0	100.0	100.0	100.0	100.0	100.0	100.0	

Values expressed as mole percent of total carbohydrate.

–, not detected.

### Strain variability within a species

For each of the three ESKAPE pathogens tested, we analyzed two independent strains, which provide a means to assess strain to strain variability by our methods. In the two *P. aeruginosa* strains, 700829 and 700888, both were found to be >85% mannose by composition, followed by minor constituents of glucose, galactose, arabinose, and rhamnose **(** **Table 1** **)**. Likewise, the major linkages of EPS exopolysaccharides from both strains in order were 2,6-Man, t-Man, 2-Man, and 3-Man **(** **Table 2** **)**. Similar to the *P. aeruginosa* results, both *A. baumannii* strains, BAA-1605 and BAA-1878, had a nearly identical composition and linkage profile not only to each other, but also to the *P. aeruginosa* strains. In sharp contrast, the two *K. pneumoniae* strains differed considerably from each other. While both strains had high mannose content, the next most abundant carbohydrates for strain 700603 were rhamnose, galactose and GlcA. For strain 700831, those residues were replaced by glucose, rhamnose, and GalA in order of importance. 700603 contained 5% GlcA, which was not found in 700831 and the latter contained trace amounts of arabinose, fucose, and xylose, which were all absent in the former. Thus, in instances where our data differ from historical results as described below, we cannot conclude whether these variations are attributable to differences in extraction/purification methods, or simply represent natural strain to strain heterogeneity within a species as displayed by the two *K. pneumoniae* strains we tested.

### Species-specific findings

Of the bacteria studied in this report, biofilms of *P. aeruginosa* are perhaps the best studied. *P. aeruginosa* produces alginate, a high molecular weight, acetylated polysaccharide that is well known for its association with the mucoid phenotype common in cystic fibrosis patients. It is composed of β-1,4 linked L-guluronic and mannuronic acids. In addition, some *P. aeruginosa* species are known to produce Psl, which consists of a repeating pentasaccharide of 3 mannose, 1 rhamnose, and 1 glucose [29], and Pel, whose exact structure is not completely known, but is reported to have a high glucose content [30], [31]. Very little data are available on the specific strains we tested, 700829 and 700888, although 700888 has been sequenced. Its genome possesses all of the genes for production of alginate (*alg44, alg8*, *algA-algZ, mucA-mucC*) and Psl (*pslA-pslM*), but does not contain the genes for Pel. Since we found the EPS exopolysaccharide from both *P. aeruginosa* strains to be predominantly mannose (∼85–90%) and the linkages were chiefly 2-Man, 3-Man, 2,6-Man, and t-Man, we conclude that the majority of the EPS exopolysaccharide we observed is consistent with Psl, although we cannot rule out additional structures.

Much is also known about exopolysaccharides of *S. epidermidis*, in particular the NRS-101 strain (a.k.a., RP62A; ATCC 35984) we employed for this study. This strain and similar *S. epidermidis* strains are known to have linear β-1,6-linked GlcNAc polysaccharides, termed the polysaccharide intercellular adhesion (PIA), encoded by the *icaADBC* operon that mediates intercellular interactions during the biofilm mode of growth [32], [33]. A separate, galactose-rich capsular polysaccharide adhesion (CPA) is also reported to be associated with this strain [34]. Christensen and colleagues used a mutant of RP62A that lacked the ability to make CPA and isolated a high molecular weight exopolysaccharide, called the slime associated antigen (SAA), which was found to be primarily glucose (∼59%) [35]. In contrast, Peters *et al* isolated a mannose-rich exopolysaccharide from a slime layer of *S. epidermidis* strain KH11 and called it the extracellular slime substance (ESS) [36]. We did not observe any evidence of PIA, which would be expected given the methods we used and the surface localization of this polysaccharide. However, our results indicate the high molecular weight EPS exopolysaccharide is 52.8% mannose and 35.7% glucose, suggesting both SAA and ESS may be present in our sample. Further experimentation is required to define the structures of these polysaccharides.

The polysaccharides of *E. coli* associated with the capsular O serogroups and K-antigens have been studied extensively, but comparatively little is known about the non-capsule high molecular weight exopolysaccharides of the EPS [37]. However, most *E. coli* strains are known to secrete colanic acid, which consists of glucose, galactose, fucose, GlcA, acetate, and pyruvate in molar proportions roughly 1∶2∶2∶1∶1∶1, respectively [38], [39]. Moreover, the O157:H7 strain we tested, ATCC 43894 (a.k.a. CDC EDL 932), has been shown to specifically generate colanic acid as its exopolysaccharide [40]. While our data show this same strain produces glucose (36.8% of total carbohydrate), fucose, (22.6%), and galactose (2.1%) as would be expected for colanic acid, the proportions are not consistent with colanic acid and most noticeably, there is a complete absence of detectable GlcA. In addition, the presence of GalNAc (26.8%), mannose (9.8%), and GlcNAc (1.9%) were unexpected findings, suggesting we isolated a previously uncharacterized exopolysaccharide.

Similar to *E. coli*, the surface exopolysaccharides (K-antigens) of *K. pneumoniae*, numbering over 80 serovars, have been studied in detail. However, there have only been a few attempts to isolate the exopolysaccharide associated with biofilm EPS. Rättö and colleagues used an ethanol extraction protocol to isolate EPS exopolysaccharide from two similar *K. pneumoniae* strains and found each contained ∼60% mannose, 20% galactose, and 17% GalA [41]. While both of our *K. pneumoniae* strains contained a high percentage of mannose as well as significant amounts of galactose and GalA, they also possessed considerable rhamnose as well as minor fractions of arabinose, fucose, GlcA, GlcNAc, and xylose, depending on strain. This indicates both strains may possess EPS exopolysaccharide structures that have not been previously characterized. Despite the remarkable heterogeneity in EPS exopolysaccharide composition between the two *K. pneumoniae* strains, it is interesting to note that the isolated exopolysaccharides bear no resemblance to the known K-antigens. For example, strain 700603 has a K6-type capsule composed of a repeating linear polysaccharide of fucose, glucose, mannose and GlcA in equal proportions [42]. In our analysis, the composition of these moieties was 0% fucose, 1.3% glucose, 49.4% mannose, and 5.0% GlcA. Thus, we can conclude that our extraction/purification methods are effective at separating the high molecular weight EPS exopolysaccharide from capsular polysaccharides.

The EPS exopolysaccharide of *A. baumannii* is the least studied of all the pathogens we tested. Cell-associated poly-β-1,6-linked GlcNAc has previously been linked to biofilm development in *A. baumannii* [43], but our protocol should not have isolated this surface polysaccharide, which is confirmed by the composition results for the two *A. baumannii* strains we tested, each of which showed only ∼1.0% GlcNAc. Crude extraction of EPS exopolysaccharide has been performed on other species of *Acinetobacter*, including *A. junii* (3 mannose: 1 galactose: 1 arabinose) [44] and *A. calcoaceticus* (4 rhamnose: 1 glucose: 1 glucuronic acid: 1 mannose) [45], but neither of these composition ratios matches the results of our two *A. baumannii* strains (BAA-1605; BAA-1878), indicating our exopolysaccharide may be a new finding.

### Mannose contribution to EPS

**Figure 2:**
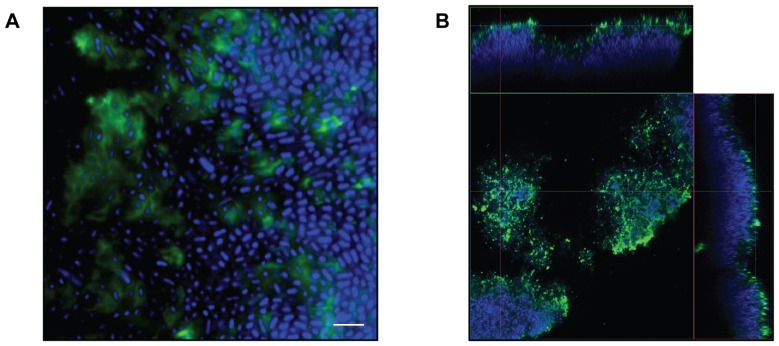
EPS staining of *E. coli* strain 43894 biofilms. The FITC-labeled mannose-specific HHA lectin was used to stain exopolysaccharides (green) and Hoechst 33342 was used to stain the bacterial nucleic acids (blue). (**A.**) Extracellular green staining of the EPS by FITC-HHA can be seen on 1 day old biofilms of *E. coli* at 200X. Scale bar  = 5 µm. (**B.**) Confocal image of 3 day old *E. coli* biofilms at 63X. The large square panel is a plan view looking down on the biofilm. The top and right-side rectangular panels are vertical sections representing the XZ plane and YZ plane, respectively, at the positions indicated by the colored lines. The biofilm is 40 µm thick (i.e Z-axis).

The most salient finding of our study was the high mannose content of the EPS exopolysaccharide across all species and strains **(** **Table 1** **)**. As such, we investigated the binding of the Amaryllis HHA lectin to pathogen biofilms. HHA specifically binds α1,3 and α1,6-linked mannose units, linkages that are common to all 8 pathogens tested **(** **Table 2** **)**. Preliminary fluorescent binding studies indicated HHA adhered to biofilms of all strains (data not shown). We therefore focused on biofilms of *E. coli* strain 43894, since the EPS exopolysaccharide of this strain had the lowest mannose content (9.8%) of all strains tested. As can be seen in **Figure 2A**, HHA binding results in an extracellular cloud around *E. coli* cells in 1 day biofilms. When 3 day biofilms are viewed by confocal microscopy **(** **Figure 2B** **)** HHA only binds to the outer surface of the ∼40 µm biofilm, presumably limited in diffusion by the density of the biofilm matrix. In contrast to the HHA lectin, which is a globular protein composed of 4 subunits, the small molecular weight nucleic acid stain penetrates the full thickness of the biofilm maxtrix, staining all *E. coli* cells within the matrix. Given the frequent use of mannose by microorganisms as a component of surface antigens or capsule, detection of mannose alone cannot be considered diagnostic for the presence of a biofilm. Nonetheless, mannose was ubiquitous in the biofilm exopolysaccharides of the five species we tested and even the EPS with the lowest mannose content was easily visualized by staining with the HHA lectin. Additional lectins or a lectin-based arrays [46] may be useful in future characterization of EPS composition from a broad range of organisms.

It has long been known that the exopolysaccharide portion of the EPS plays a substantial role in bacterial adherence and resistance [6], [7], [47] and the mannose results above suggests there may be commonalities between biofilm EPS that can be exploited for diagnostic and/or therapeutic purposes. For example, breaking the most common bonds that connect polysaccharide residues in the EPS could be an effective means of dispersing biofilms and making the subjacent bacteria more susceptible to treatment by antibiotics. Notably, bacteriophage have co-evolved with natural biofilms of host organisms for billions of years and have developed enzymatic domains on tail fiber and tailspike proteins that degrade polysaccharides of the EPS and capsule, allowing the phage access to surface receptors for infection [48]–[50]. These enzymes, generically termed depolymerases, have been shown to degrade exopolysaccharides even in the absence of phage [51], although extensive analysis of their specificity and utility as anti-biofilm agents has yet to be elucidated.

Another example of a potential anti-biofilm enzyme is dispersin B, a β-1,6-*N*-acetyl-glucosaminidase from the bacterium *Actinobacillus actinomycetemcomitans*. First described in 2003 [52], this enzyme has the ability to degrade EPS from *Actinobacillus* [4] and *S. epidermidis* [53], [54] biofilms. While enzymatic digestion of the EPS exopolysaccharide is not expected to directly kill bacterial cells, the dissolution of biofilms or prevention of future biofilm formation should allow bacteria to become re-sensitized to antibiotics and immune system mechanisms (i.e., complement, antibodies, phagocytes, etc.) Alternatively, these agents could be used to prevent biofilm formation. For example, dispersin B has been successfully incorporated into a polyurethane material, showing that materials, such as medical implants, could be engineered with anti-biofilm enzymes to prevent colonization [55].

Obtaining detailed structural characteristics by NMR for the current set of EPS exopolysaccharides in relationship to the composition and linkage data generated here will help validate our extraction and purification methods. Further characterization of ESKAPE biofilm EPS by the methods we employed here may also enable the identification and design of more effective anti-biofilm therapeutic agents.
